# Rabies in medieval Persian literature – the Canon of Avicenna (980–1037 AD)

**DOI:** 10.1186/2049-9957-3-7

**Published:** 2014-02-17

**Authors:** Behnam Dalfardi, Mohammad Hosein Esnaashary, Hassan Yarmohammadi

**Affiliations:** 1 Student Research Committee, Shiraz University of Medical Sciences, Shiraz, Iran; 2 Research Office for the History of Persian Medicine, Shiraz University of Medical Sciences, Shiraz, Iran

**Keywords:** Avicenna, Canon of medicine, Medieval history, Persia, Rabies

## Abstract

Ibn Sina (980–1037 AD), known by his full name Abu Ali al-Hussain ibn Abdallah ibn Sina and the Latin name ‘Avicenna*’*, was a Persian scholar who is primarily remembered for his contributions to the science of medicine. He authored *Al-Qanun fi al-Tibb* (*The Canon of Medicine*). Sections of his work are devoted to detailed descriptions of a number of infectious illnesses, particularly rabies. Avicenna described rabies in humans and animals and explained its clinical manifestations, route of transmission, and treatment methods. In this article, our goal is to discuss Avicenna’s 11^th^-century points of view on rabies and compare them with modern medical knowledge.

## Multilingual abstracts

Please see Additional file 1 for translations of the abstract into the six official working languages of the United Nations.

## Introduction

Rabies is an acute, progressive, and fatal anthropozoonotic infection of the central nervous system caused by viruses from the genus *Lyssavirus* and the family *Rhabdoviridae *[1]. Its history dates back thousands of years, yet despite this long history, it remains a challenge for modern medicine [2,3].

No doubt the present-day knowledge of rabies is indebted to a chain of theories and experiences developed over time. Scholars, such as Democritus (460–370 BC), Aristotle (384–322 BC), Pliny the Elder (23–79 AD), Galen (130–200 AD), Celsus (25 BC–50 AD), Rufus of Ephesus (80–150 AD), Oribasius (320–400 AD), and Aëtius of Amida (502–575 AD) are among the first physicians to specifically study rabies and contribute to the process of building up a knowledge base about it [4,5]. For instance, Aristotle noted the possibility of rabies transmission from an infected animal to a healthy one through a bite. As another example, Celsus coined the term “hudrophobia” (hydrophobia) and suggested that the saliva of a rabid animal contains a poisonous agent [6].

Some centuries later, Persian scientists, such as Rhazes (865–925 AD), Al-Akhawayni Bukhari (?–983 AD), Avicenna (980–1037 AD), and Jurjānī’ (1042–1137 AD), made significant contributions to the art of medicine, leading to the formation of ‘The Golden Age of Islam’ (ninth to 12^th^ centuries AD), an era of great development for the science of medicine in Islamic civilizations [7,8]. These scholars played key roles in gathering and preserving the extant medical knowledge of their predecessors and disseminating it to future generations. In addition, they added their own accumulated information and experiences to previous knowledge [9].

The disease of rabies and its various aspects are topics discussed in detail by Persian scientists in their works [10]. Keeping in mind that Avicenna played a leading role in the medicine of his own time, as well as in successive centuries, this paper aims to review his opinions on rabies.

## Review

### Rabies: a short historical perspective

Based on evidence found in ancient Greek and Chinese manuscripts, man has known since ancient times that rabies is a hazardous human illness related to dogs and, to some extent, cats [11]. For example, the pre-Mosaic *Eshnunna Code* of the ancient Mesopotamian civilization, which is approximately 4,000 years old, contains early indications regarding the outcome of being bitten by a mad or vicious dog [5,12].

In addition to the disease of rabies itself, its name and the term used for its etiologic agent have historical backgrounds. The modern word “rabies” is believed to be a derivation of the Latin term *rabere* (meaning: to rave) or the Sanskrit word *rabhas* (meaning: to rage) [13]. Moreover, the virus causing this disease, i.e. *Lyssavirus*, traces its name back to the two terms *Lytta* and *Lyssa*. Folklore has it that ancient people believed this disease was etiologically caused by a worm, called lytta, under the tongue [11]. It is also noteworthy, based on molecular examinations, that most likely between 888 and 1459 years ago, Lyssa viruses switched from the *Chiroptera* order to the *Carnivora* one [14].

In the 16^th^ century, Girolamo Fracastoro (1478–1553), an Italian physician, introduced breaks in human skin by an animal bite as an essential event for the occurrence of rabies [15]. Despite the long history of rabies, it was until 1769 that John Morgagni (1735–1789) (the father of pathological anatomy) theorized that the rabies virus spread itself through nerve fibers, not through veins [6]. Later, Samuel Argent Bardsley (1764–1851) theorized about the contagious nature of rabies [15]. In 1804, for the first time in the history of medicine, Georg Gottfried Zinke (1771–1813) proved the infectious nature of rabies [16]. In 1821, Francois Magendie (1783–1855), a French neurophysiologist, recognized the transmission of this disease to dogs by means of inoculation with a rabid human’s saliva [15,17]. Some years later, in 1885, Louis Pasteur (1822–1895) developed the first successful vaccine against it [16]. Then, in 1903, Adelchi Negri (1876–1912), an Italian pathologist and microbiologist, provided the first descriptions of virus-nerve cell interaction in the brains of rabies-infected animals. He detected cytoplasmic bodies (today called Negri bodies) in this group of nerves [18,19]. About six decades later, in 1962, Sokolow and Vaney demonstrated that Negri’s cytoplasmic bodies are in fact RNA granules embedded in a matrix of DNA [15].

### Avicenna and the *Canon of Medicine*

**Figure 1:**
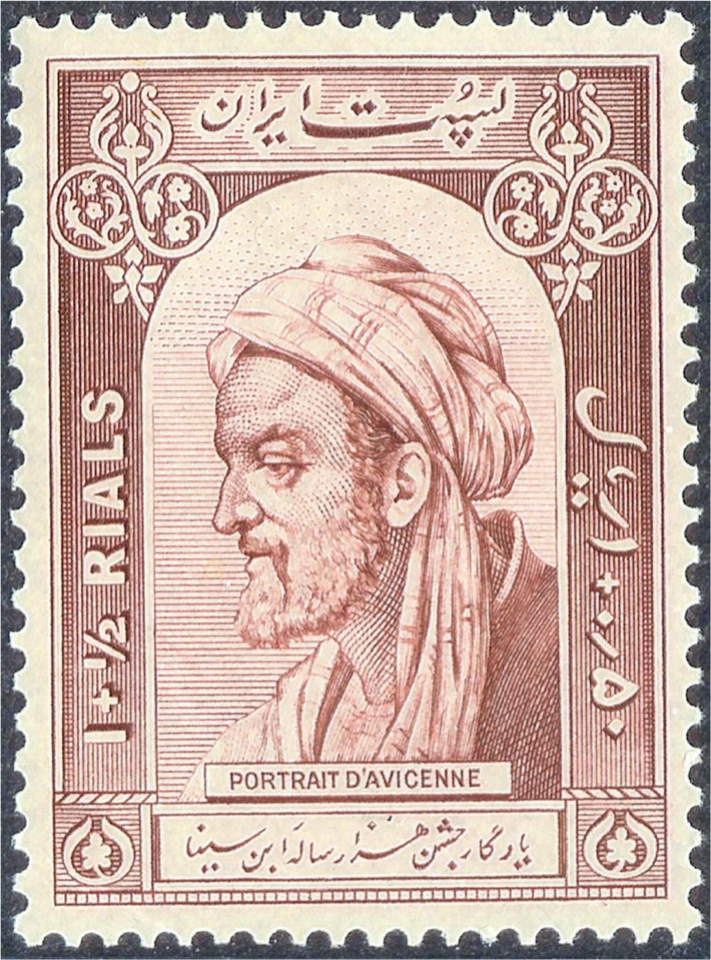
A portrait of Avicenna on an Iranian postage stamp.

**Figure 2:**
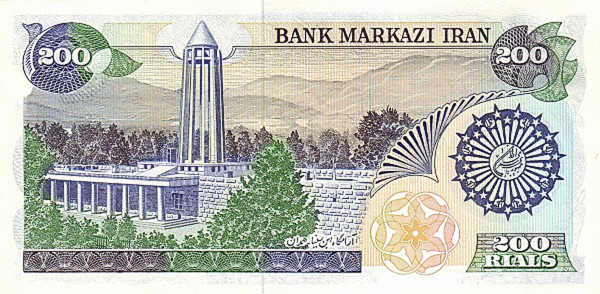
**The tomb of Avicenna on an Iranian banknote.** This tomb is located in Hamadan, Iran.

Abu Ali al-Hussain ibn Abdallah ibn Sina (980–1037 AD), who is commonly known as Avicenna in western literature, was an 11^th^-century Persian scientist renowned throughout history for his significant contributions to the art of medicine (see Figures 1 and 2) [20].

Avicenna was born in 980 AD. His lifetime coincided with the Islamic Medicine Golden Age (ninth to 12^th^ centuries AD). Undoubtedly, his contributions had a great impact on the development of medicine in this significant time period [21].

**Figure 3:**
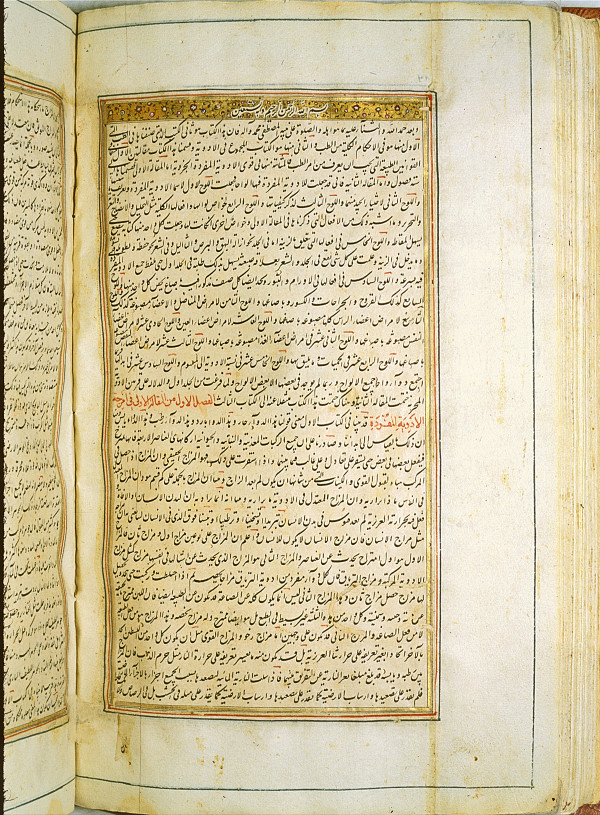
**Opening of the second book of the *Al-Qanun fi al-Tibb *(*The Canon of Medicine*) (from the early 15**^**th **^**century, probably Iran).** (Courtesy of the US National Library of Medicine).

The field of healing arts was a main focus of Avicenna’s interest and attention [22]. Among his extant works, the *Al-Qanun fi al-Tibb* (*The Canon of Medicine*), an encyclopedia originally written in Arabic, serves as Avicenna’s main work related to medicine (see Figure 3) [23,24]. This textbook remained the standard reference for medical education in the West up until the 16^th^ century and in Middle Eastern countries until the 19^th^ century [21,25]. Avicenna started composing the Canon in about 1012 AD. He completed it between 1020 and 1025 AD, after a lengthy period during which he traveled to different cities in Persia and gained new experiences and knowledge [20,24]. The Canon is claimed to be the most influential textbook of medicine ever written; it is honored as *The Medical Bible of the Middle East *[22,26].

The Canon consists of five books, each covering specific medical topics. The first volume is devoted to general issues and principals relating to medicine; the second volume primarily provides descriptions of several simple drugs, compiled alphabetically; the third volume deals with various diseases involving body organs (from head to foot) and their different clinical findings and managements; the fourth volume presents diseases that affect more than a single part of the body, such as fevers; and the final volume explains recipes for compound remedies [23,27]. In writing this textbook, Avicenna gathered the major medical information available from some of his great predecessors, such as Galen and Rhazes, and merged them with his own clinical experience. He sometimes criticized his predecessors [27].

Avicenna’s undeniable contributions to the science of medicine resulted in the medical community admiring him with and bestowing him with names such as The Galen of Islam, The Prince and Chief of Physicians, The Teacher Second Only to Aristotle, and The Aristotle of Arabs [22,23].

### Insights into Avicenna’s knowledge of rabies

Rabies is an infectious disease discussed fully by Avicenna treatises, entitled “*Fi Sefati al-Kalbi al-Kalb wa al-Ze’bi al-Kalb wa ibn Avi al-Kalb*” *(“On Description of Rabid Dog and Rabid Wolf and Rabid Jackal/Weasel”*) and “*Fi Ahvali Min Azahi al-Kalbi al-Kalb*” (“*On Conditions Arising from the Bite of a Rabid Dog”*), included in the fourth section of the sixth part of the fourth volume of the Canon [28].

In Avicenna’s opinion, rabies primarily results from an imbalance of the four humors within dogs’ bodies. He explains in detail the manifestations of rabies in dogs, including fear of water, excessive salivation, and aggressive behavior.

Avicenna points to the transmission of rabies to healthy animals and humans through bites from a rabid animal or human. For instance, he presents a case of animal-to-human transmission of rabies following the bite of rabid animal, as follows: “… *A rabid mule bit its owner; its owner became insane in the way of a dog when it becomes rabid*…”. He noted the occurrence of rabies in other animals such as foxes, weasels, jackals, and wolves.

Avicenna, according to the time of presentation, describes clinical manifestations of rabies in humans. These clinical findings include: pain at the site of the bite (similar to the pain resulting from other ulcers), nightmare, aggression and agitation, confusion and loss of thinking ability, talking to self, thirst, fear of crowded places (corresponding to agoraphobia in current terminology), fear of light (equivalent to photophobia in modern nomenclature), spasm of fingers, redness of extremities and face, presentation of purulent ulcers on face, increase in wound pain, hoarseness, fear of water (hydrophobia in current nomenclature) seizure, and death. Avicenna explains that patients with rabies may experience severe thirst but often refuse to drink. On the contrary, those patients who drink water are unable to swallow it.

According to Avicenna’s statements, in the late stages of the disease (after presentation of hydrophobia) the patient has no chance of treating the disease. In his opinion, the time in which rabies will lead to death ranges from seven days to six months (with an average of 40 days). Avicenna explains that patients who experience more severe and immediate blood loss from a bite wound have a better prognosis.

Of note, Avicenna points to the possibility of the transmission of rabies through saliva. He states: “*Everyone who eats the remaining part of his [a rabid human’s] food or drinks his water becomes rabid.*”

To prevent contracting rabies after being bitten by a rabid dog, Avicenna prohibits the closure of wounds caused by the bite for 40 days. Cupping and sucking the site of the bite (immediately after the bite occurs) are two of his other recommendations. According to Avicenna’s statements, it is necessary to evaluate the manifestations of rabies in dogs which are responsible for the bite.

Avicenna prescribes different types of topical medications, which he states can be useful in the treatment process of rabid patients. Among these are ointments which take in and reduce the effect of the rabies’ toxin, ointments which prevent the closure of a bite wound, etc.

Two of Avicenna’s statements about the treatment of rabies are remarkable. He introduces rabid dogs’ blood as an antitoxin for a rabid animal’s bite. Avicenna also says: “*They [previous physicians] said and several physicians confirmed that it would be very useful if you put the liver of a rabid dog on the wound resulting from the bite of a rabid dog*” [27].

Today, it is known that the rabies antibody is present in the blood of rabid dogs. In modern medicine, in addition to systemic usage, the human rabies immunoglobulin is used for local injection in the margins of the bite wound. This results in passive immunization and will reduce post-exposure mortality [29]. Considering this fact, Avicenna’s explanation can be regarded as early recognition of today’s known post-exposure passive immunization.

Moreover, today it is evident that rabid animals produce virus-laden saliva [29]. Avicenna pointed to the possibility of transmission of rabies through the saliva of infected humans approximately eight centuries before Francois Magendie’s studies and Georg Gottfried Zinke’s findings on this issue.

## Conclusions

The medieval Persian scientist Avicenna provided a meticulous description of the manifestations, course, prognosis, and treatment of the rabies disease. In spite of some of his extraneous advice, the main part of his explanation shows similarities to modern medicine. Early in the history of medicine, Avicenna reported the transmission of rabies through saliva. He provided an early description of the present-day method of post-exposure passive immunization. It can be said that Avicenna made a significant contribution to the medieval Persian knowledge of the disease of rabies.

## Competing interests

The authors declare that they have no competing interests.

## Authors’ contributions

MHE drafted Part 1. BD, MHE, and HY drafted Part 2. BD and HY drafted Part 3. BD and HY drafted Part 4. All three authors edited the entire manuscript and approved the final version.

## Supplementary Material

Additional file 1 Multilingual abstracts in the six official working languages of the United Nations. Click here for file
