# Supplementary material for: Rabies in medieval Persian literature – the Canon of Avicenna (980–1037 AD)
Source: Infect Dis Poverty. 2014 Feb 17;3:7. doi: 10.1186/2049-9957-3-7 (PMC3933285; doi:10.1186/2049-9957-3-7)
Supplement: Additional file 1 — Multilingual abstracts in the six official working languages of the United Nations. [file 2049-9957-3-7-S1.pdf]

Translation of the abstract into the six official working languages of the United Nations

مرض السعار بالنشرات الطبية الفارسية- قانون ابن سينا (980-1037 م)

بهنام دلفاردی، محمد حسین اثنی عشری، حسن یارمحمّدی

ملخص

أبو علي الحسين بن عبد الله ابن سينا والمعروف بابن سينا (980-1037م) واسمه اللاتين (**Avicenna**) هو معلم فارسي معروف باسهاماته في مجال العلوم الطبية. ألف كتاب القانون في الطب، وخصص فصول من الكتاب لوصف عدد من الأمراض المعدية ومنها السعار على وجه الخصوص. قدم ابن سينا وصفًا للسعار عند البشر والحيوان وشرح أعراضه الطبية ومسار نقل العدوى وطرق العلاج. نهدف من هذا البحث مناقشة وجهة نظر ابن سينا لداء السعار بالقرن الحادي عشر ومقارنتها بالعلوم الطبية الحديثة.

Translated from English version into Arabic by Laila Mostafa, through

TRANSLATORS WITHOUT BORDERS

中世纪波斯文化中的狂犬病-阿维森纳学说（公元 980-1037 年）

贝南·达尔法尔迪；穆罕默德·侯赛因·艾斯纳沙瑞；哈桑·亚莫哈买迪

摘要

伊本·西纳（伊斯兰经名全名为 Abu Ali al-Hussain ibn Abdallah ibn Sina），拉丁名字为阿维森纳，生于公元 980 年，卒于 1037 年，是中世纪波斯在医学方面最著名的学者。医学百科全书式的著作《医典》（*Al-Qanun fi al-Tibb*）即为阿维森纳所著，书中的部分章节对一些传染性疾病尤其是狂犬病进行了详细的描述。阿维森纳描述了人及动物狂犬病，并解释了其临床表现、传播途径和治疗方法。本文的目的是讨论阿维森纳 11 世纪针对狂犬病的观点，及其与现代医学理论的比较。

Translated from English version into Chinese by Wang Li-hua, through

La rage dans la littérature médiévale persane – Le Canon d'Avicenne (980–1037 apr. J.-C.)

Behnam Dalfardi; Mohammad Hosein Esnaashary; Hassan Yarmohammadi

Résumé

Ibn Sina (980–1037 apr. J.-C.), de son nom complet Abu Ali al-Hussein ibn Abdallah ibn Sina et appelé « Avicenne » en latin, était un érudit perse avant tout célèbre pour ses contributions en matière de médecine. Il est l'auteur de l'ouvrage *Al-Qanun fi al-Tibb* (*Le Canon de Médecine*). Certaines parties de son œuvre présentent des descriptions détaillées d'un certain nombre de maladies infectieuses, dont la rage. Avicenne y décrit les effets de la rage parmi les êtres humains et les animaux et explique ses manifestations cliniques, ses moyens de transmission et les méthodes de traitement. Dans le cadre de cet article, nous nous attachons à aborder les points de vue d'Avicenne sur la rage, qui sont ceux d'un homme du 11ème siècle, et à les comparer aux connaissances médicales modernes.

Translated from English version into French by Eric Ragu, through

TRANSLATORS
WITHOUT BORDERS

Бешенство в средневековой персидской литературе. Канон Авиценны (980–1037 н.э.)

Бенхам Далфарди; Мохаммед Хоссейн Эсншари; Хассан Ярмохаммади

Краткое изложение

Ибн Сина (980-1037 н.э.), известный под своим полным именем Абу Али ал-Хуссейн ибн Абдаллах ибн Сина и латинским именем Авиценна, был персидским ученым, наиболее знаменитым своими вкладами в науку медицины. Он написал *Al-Qanun fi al-Tibb* («Канон врачебной науки»). Отдельные части этой работы посвящены подробному описанию различных инфекционных заболеваний, в частности, бешенства. Авиценна описал бешенство в человеке и животных и объяснил его клинические проявления, путь передачи и способы лечения. В данной статье нашей целью является рассмотрение точки зрения Авиценны (XI век) на бешенство и сравнение ее с современными медицинскими знаниями.

Translated from English version into Russian by Elena McDonnell, through

TRANSLATORS
WITHOUT BORDERS

La Rabia en la literatura medieval persa – El Canon de Avicena (980–1037 AD)

Behnam Dalfardi; Mohammad Hosein Esnaashary; Hassan Yarmohammadi

Abstracto

Ibn Sina (980–1037 AD), conocido por su nombre completo Abu Ali al-Hussain ibn Abdallah ibn Sina y el nombre latín 'Avicena', fue un sabio persa que es principalmente recordado por sus contribuciones a la ciencia de la medicina. Es el autor de *Al-Qanun fi al-Tibb* (*El Canon de Medicina*). Secciones de este trabajo están dedicadas a detalladas descripciones de una serie de enfermedades infecciosas, particularmente la rabia. Avicena describió la rabia en humanos y animales, y explicó sus manifestaciones clínicas, vías de transmisión, y métodos de tratamiento. En este artículo, nuestro objetivo es discutir los puntos de vista del siglo XI de Avicena respecto de la rabia y compararlos con el conocimiento médico moderno.

Translated from English version into Spanish by Denise Tarud, through

TRANSLATORS WITHOUT BORDERS
